# Polyaniline/palladium nanohybrids for moisture and hydrogen detection

**DOI:** 10.1186/s13065-018-0461-y

**Published:** 2018-08-16

**Authors:** Chanaka Sandaruwan, H. M. P. C. K. Herath, T. S. E. F. Karunarathne, S. P. Ratnayake, G. A. J. Amaratunga, D. P. Dissanayake

**Affiliations:** 1 Sri Lanka Institute of Nanotechnology (SLINTEC), Homagama, Sri Lanka; 2 Department of Chemistry, University of Colombo, Colombo 3, Sri Lanka; 3 Department of Engineering, University of Cambridge, Trumpington Street, Cambridge, CB2 1PZ UK

**Keywords:** Conductive polymers, Nanoparticles, Sensors, Impedance spectroscopy

## Abstract

Palladium nanoparticles display fascinating electronic, optical and catalytic properties, thus they can be used for various applications such as sensor fabrication. Conducting polymers such as polyaniline have also been widely used in sensor technology due to its cost effectiveness, versatility, and ease of synthesis. In this research, attention was given to unify the exceptional properties of these two materials and construct palladium nanoparticle coated polyaniline films to detect hydrogen and moisture. Electrochemical polymerization of aniline was carried out on gold sputtered epoxy resin boards. Polyaniline film was generated across a gap of 0.2 mm created by a scratch made on the gold coating prior to electrochemical polymerization. A palladium nanoparticle dispersion was prepared using sonochemical reduction method and coated on to polyaniline film using drop-drying technique. Polyaniline only films were also fabricated for comparative analysis. Sensitivity of films towards humidity and hydrogen was evaluated using impedance spectroscopy in the presence of the respective species. According to the results, polyaniline films exhibited an impedance drop in the presence of humidity and the response was significantly improved once palladium nanoparticles were incorporated. Interestingly, polyaniline only films did not respond to hydrogen. Nevertheless, palladium nanoparticle coated polyaniline films exhibited remarkable response towards hydrogen.

## Introduction

Hydrogen gas plays a significant role in green energy technology as it is known as the “common fuel of the future”. Being a clean, renewable and efficient fuel, it holds a commendable usability as a green energy source [1, 2]. Currently, it is utilized in many industries such as petroleum refining and metallurgical engineering [2–7]. Hydrogen has distinctive properties such as low minimum ignition energy, wide flammable range and detonation sensitivity. It is a colorless, odorless and a tasteless gas. Due to these reasons, detection of hydrogen is highly important [1, 3]. Different types of sensors can be used to detect hydrogen qualitatively or quantitatively. These sensors can be categorized as catalytic, thermal, electrochemical, mechanical, optical, acoustic and conductive sensors [3, 8, 9]. In this regard, palladium nanoparticles (Pd NPs) have been used extensively to sense hydrogen [3, 7–16], due to its special properties at the nanoscale and its affinity towards H_2_ [10, 11, 17–31]. During the sensing of H_2_, adsorption of H_2_ on to the surface of the Pd NPs causes the α-phase (conductive) of PdH_x_ to convert to the β-phase (less-conductive) which leads to the detection of hydrogen [13–15].

Humidity which is simply the water vapor in air can be expressed in terms of absolute humidity (ppm), dew/frost point (D/F PT) and relative humidity (RH) [32, 33]. Humidity plays a significant role in automated industrial processes such as pharmaceutical production, food processing, electronics fabrication and agriculture [32, 34–37] hence, it is essential to monitor, detect and control such parameter [33]. Humidity can be measured using different types of sensors which are categorized as capacitive, resistive and thermal conductive sensors [32–35, 38–49], which are primarily based on the measurement of RH. Humidity sensing action of polyaniline (PAni) is attributed to the changes of resistance due to the adsorption of water molecules to its surface. Exposure to water vapor protonates PAni (acid–base reaction) via electron hopping assisted by a proton transfer mechanism and shows increased conductivity [43].

Detection of both hydrogen/humidity together is quite a challenge as the sensing system should encompass high sensitivity, wide dynamic range, good stability and quick response capability [3, 8, 50–52]. Even though researchers have used palladium nanoparticles for the detection of hydrogen and polyaniline conductive polymers for the detection of humidity, a combined system has not been investigated up to date. Hence, in this study, both hydrogen and humidity sensing ability of Pd nanoparticle coated PAni thin film have been investigated.

## Materials and methods

### Materials

All chemicals and reagents used in this study were analytical grade and purchased from Sigma-Aldrich, USA. Aniline was double distilled prior to electrochemical polymerization and all other chemicals were used as received. All aqueous solutions were prepared using distilled water.

### Preparation of gold sputtered glass–epoxy resin substrate for electrochemical deposition of PAni

Initially, copper clad boards (1.0–1.5 mm thick) containing an epoxy resin (ER) were cut into 1 × 4 cm size chips using a laser cutter. Then, a thin marker pen line (0.2 mm) was drawn on its longest axes of cemetery on the copper plated side. Resulting chip was then treated with previously prepared FeCl_3_·6H_2_O solution to remove copper plating on the unmarked area. Etched chip was then treated with acetone and ethanol to remove the pen line which was drawn before and to acquire a thin copper line. This copper line-containing chip was gold sputtered (Hitachi E1010) under a vacuum of approximately 10 Pa and a discharging current of 10 mA up to 120 S. A small scratch was made on top of the copper line to remove gold coating to clear the thin copper line. Obtained chip was again treated with previously prepared FeCl_3_·6H_2_O to remove the thin copper line to obtain two gold electrodes separated by 0.2 mm gap.

### Synthesis of PAni thin film deposited ER for humidity sensing

Prepared gold sputtered ER containing two separate gold electrodes was then dipped in a solution containing 4.20 g of double distilled aniline in 100.0 cm^3^ of 0.5 M H_2_SO_4_. The two gold electrodes were then connected together using a crocodile clip and connected to the positive terminal of the power supply and a voltage of 1.41 V was applied for 25 min. Another gold sputtered ER electrode was used as the counter electrode. The solution mixture was stirred at a rate of 100 rpm during the electrochemical polymerization. This procedure generated a thin polyaniline layer between the separated gold electrodes making an electrical contact.

### Synthesis of Pd nanoparticle dispersion

Firstly, Pd(NO_3_)_2_ (5.0 g) was dissolved in 50 ml of water. Then, the reaction mixture was prepared by adding 0.2 g of Poly(vinylpyrrolidone) (PVP, M.W-10,000) into ethylene glycol (40 ml) and mixed for 15 min [53]. Then 2.0 ml of previously prepared Pd(NO_3_)_2_ solution (8.7 × 10^−4^ mol) was added to the reaction mixture. Finally, this reaction mixture was subjected to continuous sonochemical irradiation for 120 min using a multiwave ultrasonic generator operating at an amplitude of 20 kHz [53].

### Preparation of Pd nanoparticles incorporated PAni thin films (PIPTF)

Resulted Pd nanoparticle dispersion was drop dried on the surface of PAni thin film using vacuum drying at 50 °C for 30 min. This was repeated 10 times and resulting chips were subjected to H_2_ and H_2_O sensing experiments.

For a comparative analysis, Pd nanoparticle dispersion was spin coated on the surface of gold sputtered ER boards.

### Morphological studies

Morphology of resulted chips was examined using scanning electron microscopy (SEM) (HITACHI SU6600) and atomic force microscopy (AFM) (PARK SYSTEMS XE100).

### Impedance measurements

A 5.0 Vpp sinusoidal signal was supplied to the sensors using a function generator (TEKTRONIX 3022B) at different humidity [54] and hydrogen environments [6, 13]. Moisture traps were used to ensure that hydrogen environments were 100% moisture free. The output voltage signals were measured using a dual channel digital oscilloscope (TEKTRONIX DPO 2012). The variations in output signals (amplitude and the phase shift) as the signal frequency varied (from 20 Hz to 25 MHz) were observed and the impedance was measured.

## Results and discussion

### Characterization of gold sputtered ER boards

**Fig. 1:**
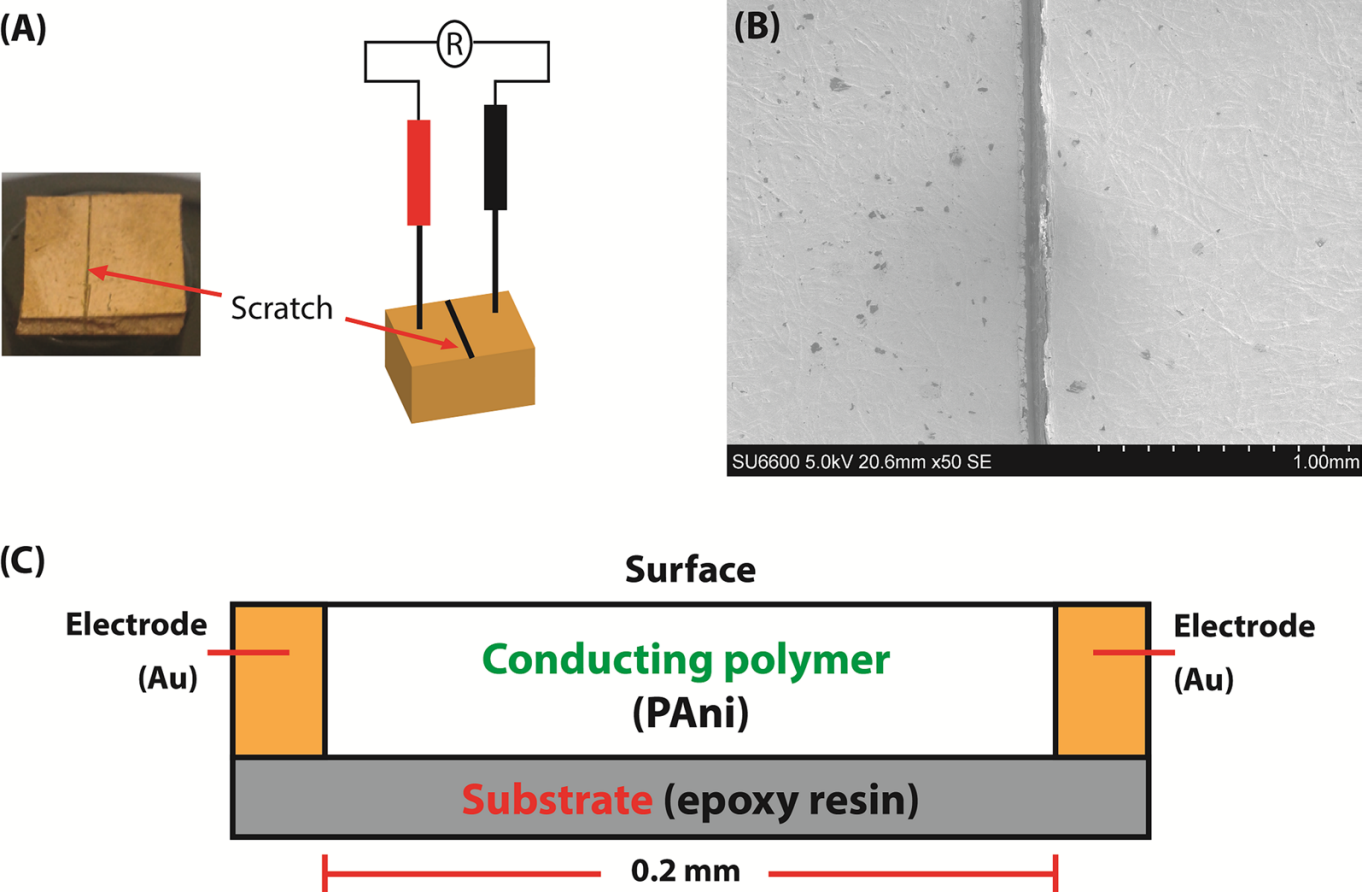
**A** Scratched gold sputtered ER boards, **B** SEM image of the scratched gold sputtered ER boards and **C** schematic diagram of the sensor

Sputtered gold film was characterized using resistance measurements, which was found to be less than 5 Ω between two 10 mm distance points. Resistivity reached infinity in between two gold electrodes after they were separated by a narrow scratch (0.2 mm) (Fig. 1A, B) and the schematic diagram of the sensor is given in Fig. 1C.

**Fig. 2:**
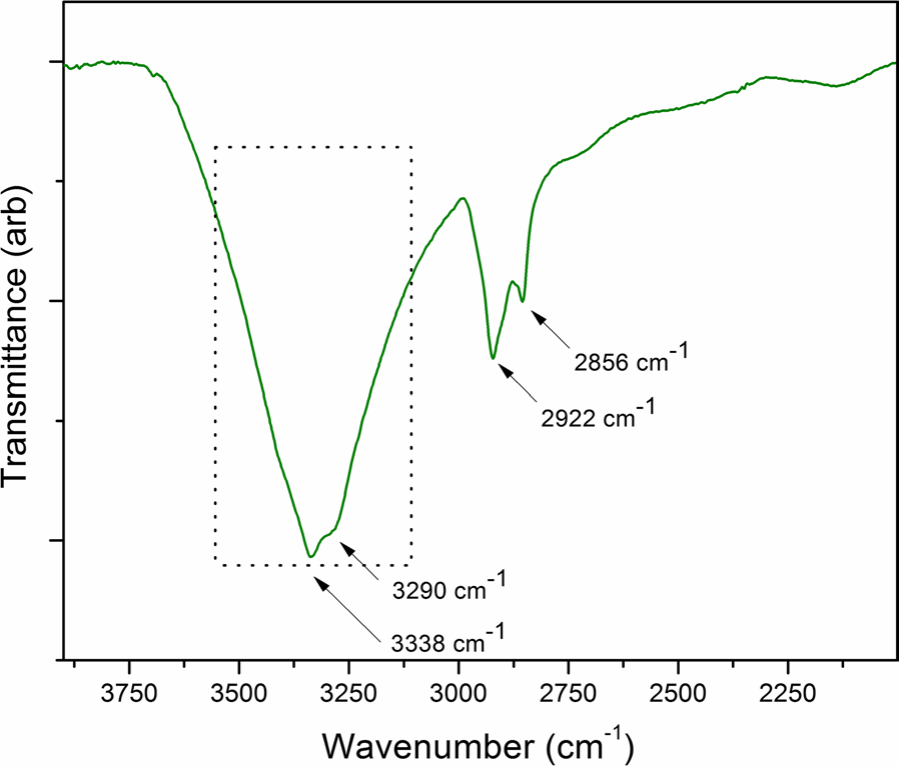
FT-IR spectrum of the surface of ER board

The strong absorption band having a distribution from 3400 to 3500 cm^−1^ region in the Fourier transform infrared (FT-IR) spectrum of the ER (Fig. 2) strongly suggest the presence of hydroxyl groups on the ER surface. These hydroxyl groups work as potential sites for the adsorption of aniline molecules during the electrochemical polymerization process [54].

### Characterization of PAni film deposited ER boards

**Fig. 3:**
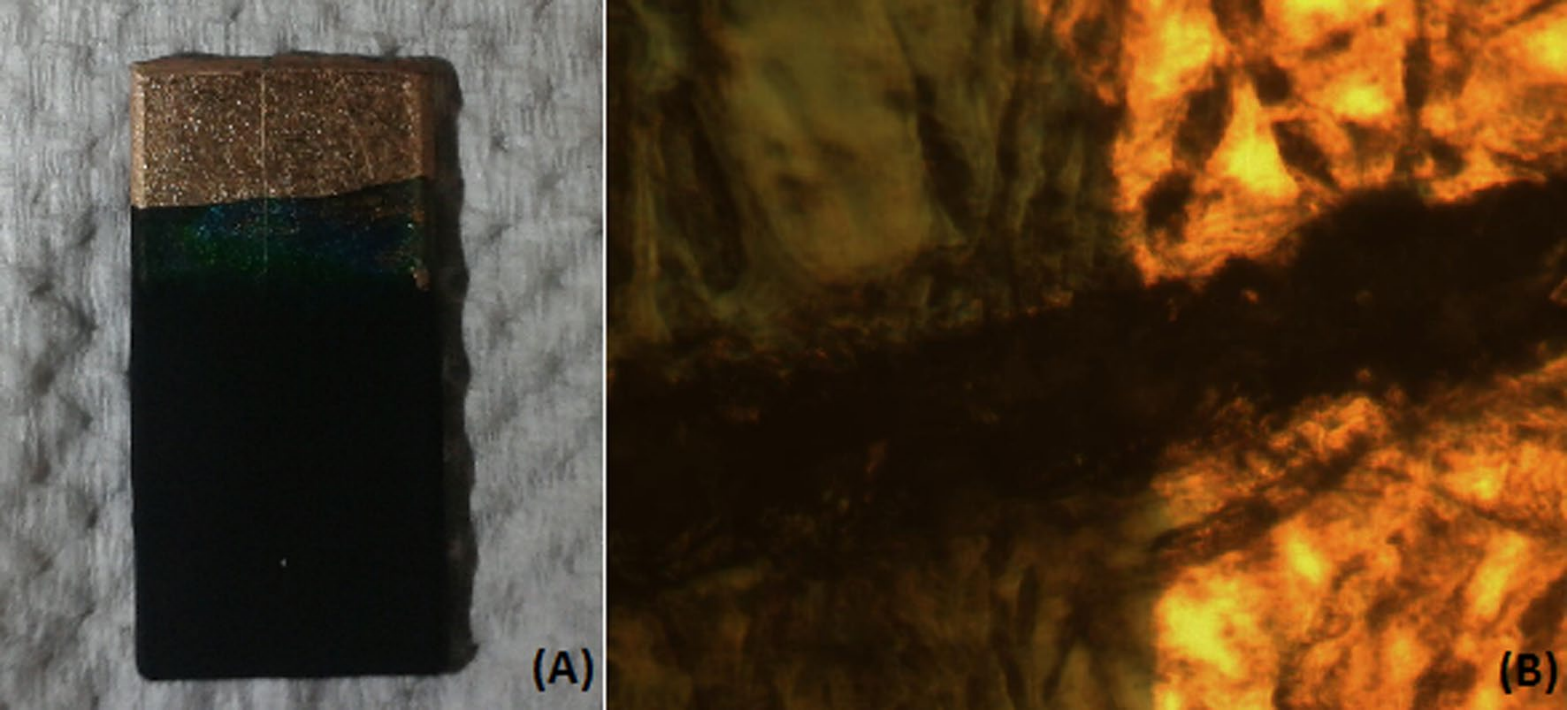
**A** PAni film deposited gold sputtered ER board and **B** optical microscopic image of PAni film deposited gold sputtered ER board

The electro-deposition of polyaniline started as a blue colored layer on the surface of the gold sputtered ER board. This is the poly pernigraniline base which is the intermediate protonated form of polyaniline [55]. Later, it becomes green as pernigraniline is converted into the final product, the protonated emeraldine form of polyaniline (Fig. 3A).

In order to deposit a uniform layer of polyaniline, it is important to maintain a low voltage during the deposition [55, 56]. Optical microscopic images revealed that the resulted two electrodes are connected by the PAni film effectively (Fig. 3B). The thickness of electro-polymerized PAni films was measured using a sensitive thickness gauge and was recorded as 42(± 1) μm.

**Fig. 4:**
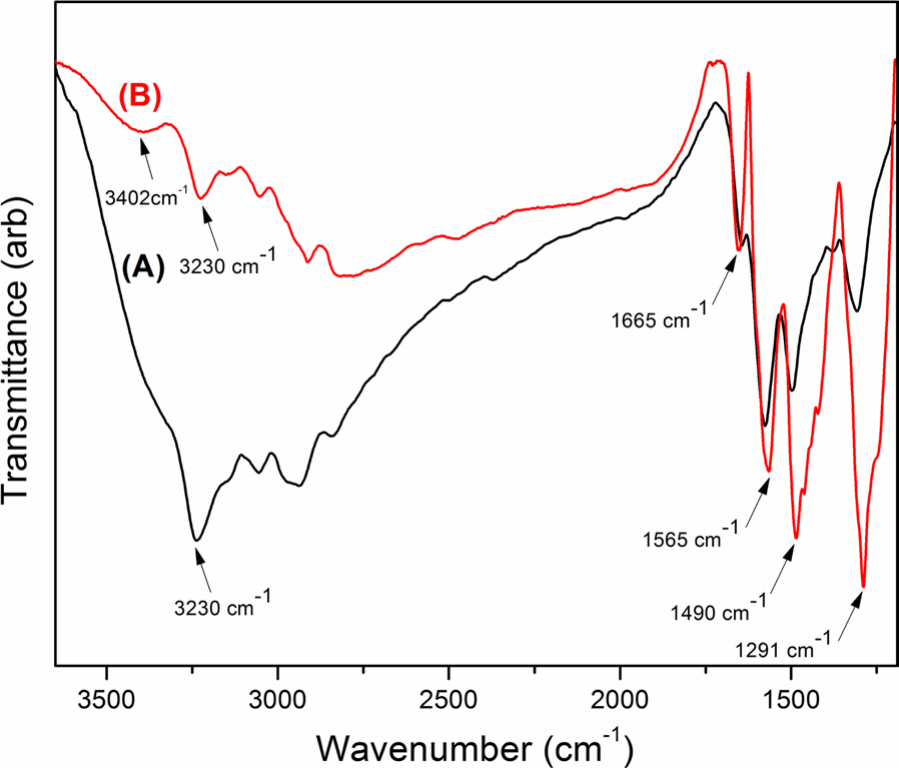
FT-IR spectra of PAni film (A) before drying process and (B) after drying process

In order to identify the chemical composition of the deposited PAni film, FT-IR spectra were recorded in the range of 4000–400 cm^−1^ before (A) and after (B) drying (Fig. 4).

A broad peak around 3400 cm^−1^ is responsible for the N–H stretching of PAni. The peak at 3230 cm^−1^ accounts for the OH stretching of water molecules physisorbed to the PAni backbone. A sharp band at 1650 cm^−1^ in PAni is due to asymmetric stretching and bending modes of water. It can be clearly seen that the broadness of the OH stretching band is reduced after the drying process. The peaks at 1565 cm^−1^ and 1490 cm^−1^ are due to the quinoid and benzoid structures of PAni, respectively. Meanwhile, secondary C–N stretching band can be observed around 1290 cm^−1^ which further confirms the presence of Quinoid and Benzoid structures of PAni [57, 58]. According to the structural analysis, the ratio between quinoid to benzoid was found to be 1. This clearly indicates the presence of highly doped emeraldine salt form of polyaniline [55].

### Characterization of Pd nanoparticle dispersion

**Fig. 5:**
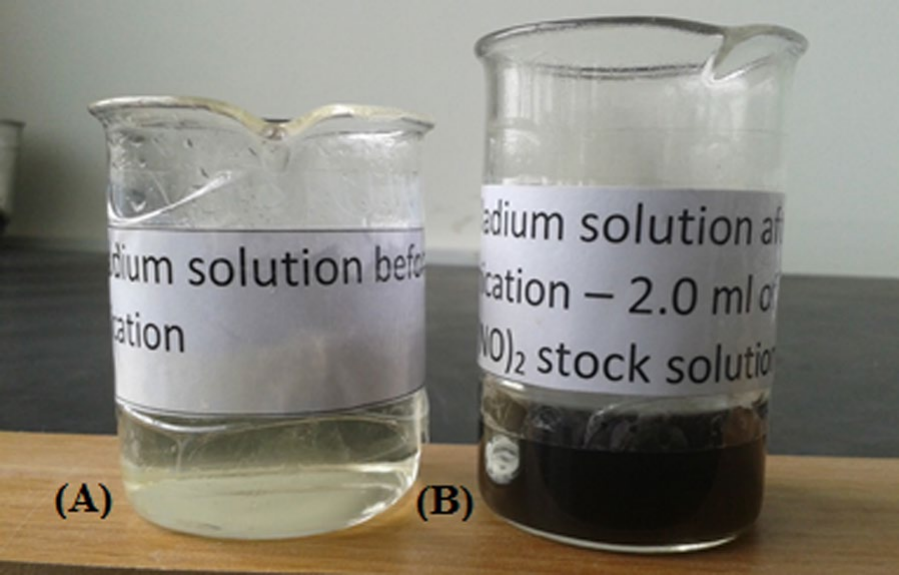
Appearance of **A** the reaction mixture before the sonication process started and **B** the reaction mixture after the sonication process is completed

The pale yellow color of the Pd(NO_3_)_2_ mixture was changed into dark brown after ultrasonication (Fig. 5). This observation provided an initial evidence for the formation of Pd nanoparticles during the sonochemical reduction of Pd(NO_3_)_2_ [53]. Resulted Pd nanoparticle solution persists over 15 months without any aggregation. The change in pH of the reaction mixture from 2.97 to 2.62 after the ultrasonication is in good agreement with the literature, confirming the reduction of Pd^2+^ ions to Pd nanoparticles [53, 59].

**Fig. 6:**
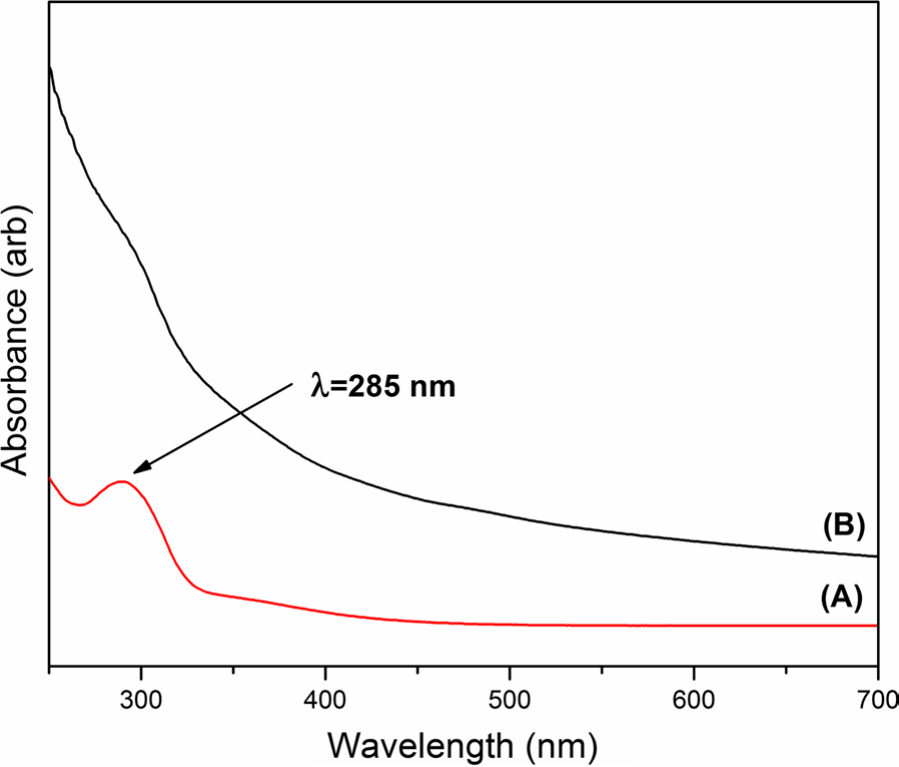
UV-Visible spectra of (A) starting solution and (B) sample after sonication

Pd nanoparticle formation was investigated using UV–Visible spectroscopy in the wavelength range of 250–750 nm. UV absorption of the Pd nanoparticle suspension after the sonication was compared with the initial solution containing ethylene glycol, PVP and Pd(NO_3_)_2_. The UV band around 290 nm due to the d–d transition in the aqua complex [Pd(H_2_O)_4_]^2+^, disappeared with the formation of Pd nanoparticles [60]. In addition, the spectrum of the ultrasonicated sample yields broad continuous absorptions in the UV–visible range which can be assigned to the presence of Pd nanoparticles (Fig. 6) [53].

**Fig. 7:**
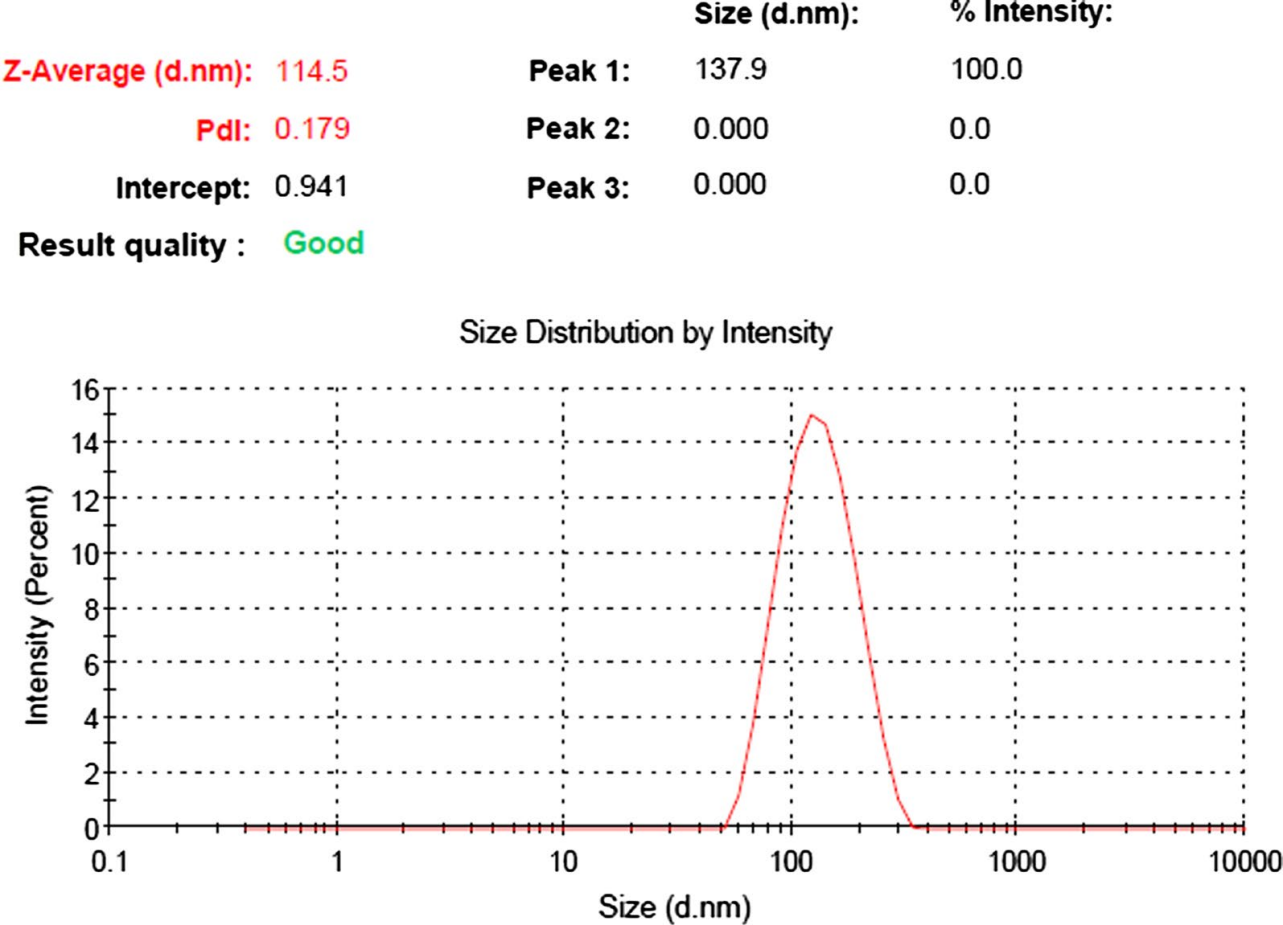
Size distribution of Pd nanoparticle dispersion

The average dynamic diameter of Pd-nanoparticles is around 115 nm (Fig. 7). The polydispersion index (PDI) is 0.179, which indicates the uniformity of the Pd nanoparticle dispersion.

**Fig. 8:**
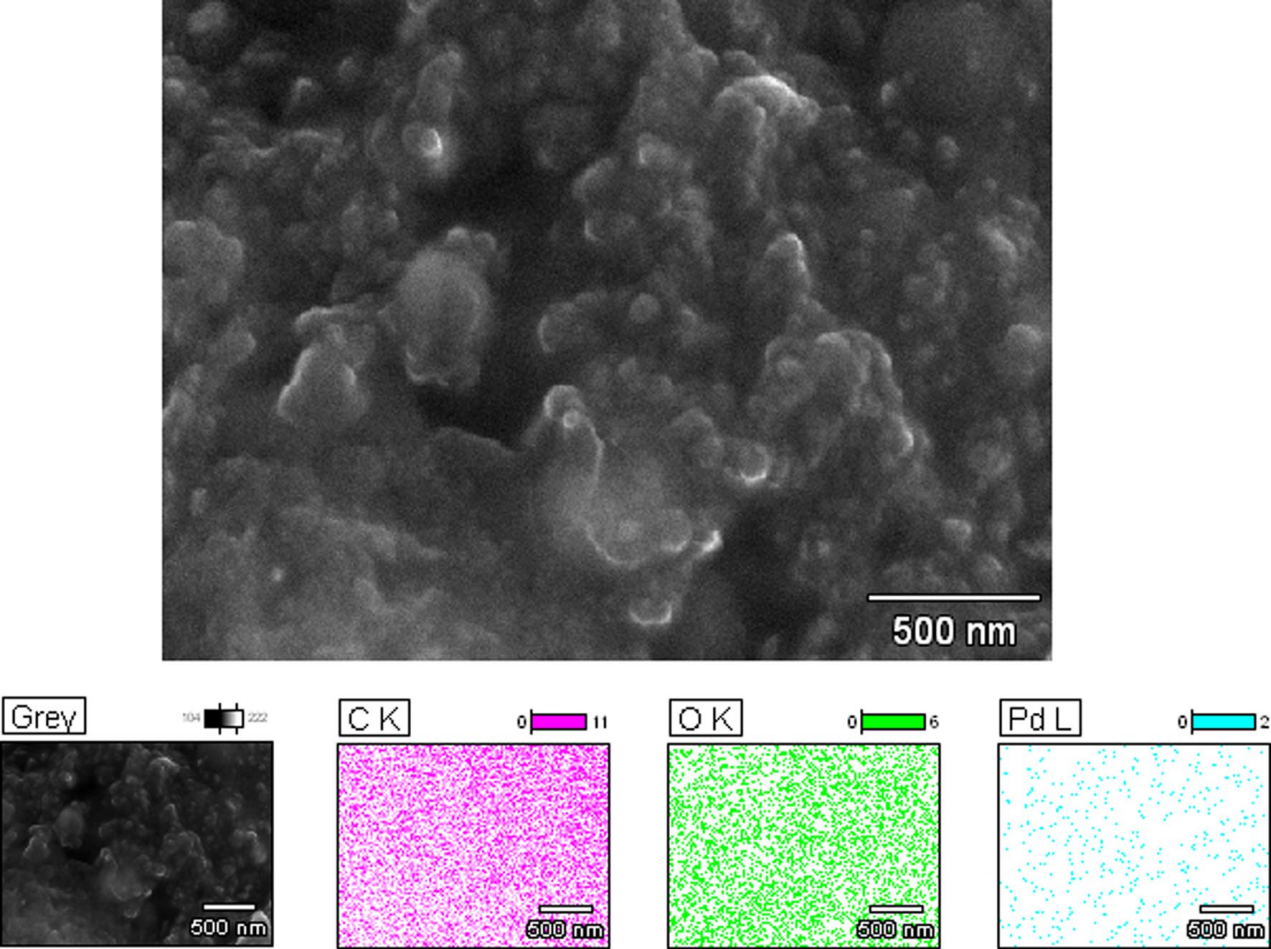
SEM image and EDX analysis of Pd dispersion

However, SEM analysis revealed that the particle size varied from 20 to 40 nm (Fig. 8). The discrepancy between SEM and dynamic light scattering based particle size measurements could be attributed to the formation of polymer–metal cluster complexes by the interaction of protective polymers and Pd nanoparticles. The particle size analyzer identifies polymer protected nanoparticle aggregates as a single unit instead of separate entities. SEM images support the formation of bulky polymer–metal complexes which (nanoparticle-buried polymer matrix) can be clearly observed [61]. In addition, a uniform distribution of the Pd nanoparticles in the polymer matrix also can be observed in the SEM images. Energy dispersive X-ray (EDX) analysis was conducted to verify the presence and to quantify the amount of Pd present in the Pd nanoparticle dispersion. Results indicated that 3.74% by weight of Pd is present in the matrix (Fig. 8).

### Characterization of Pd nanoparticles incorporated PAni films

**Fig. 9:**
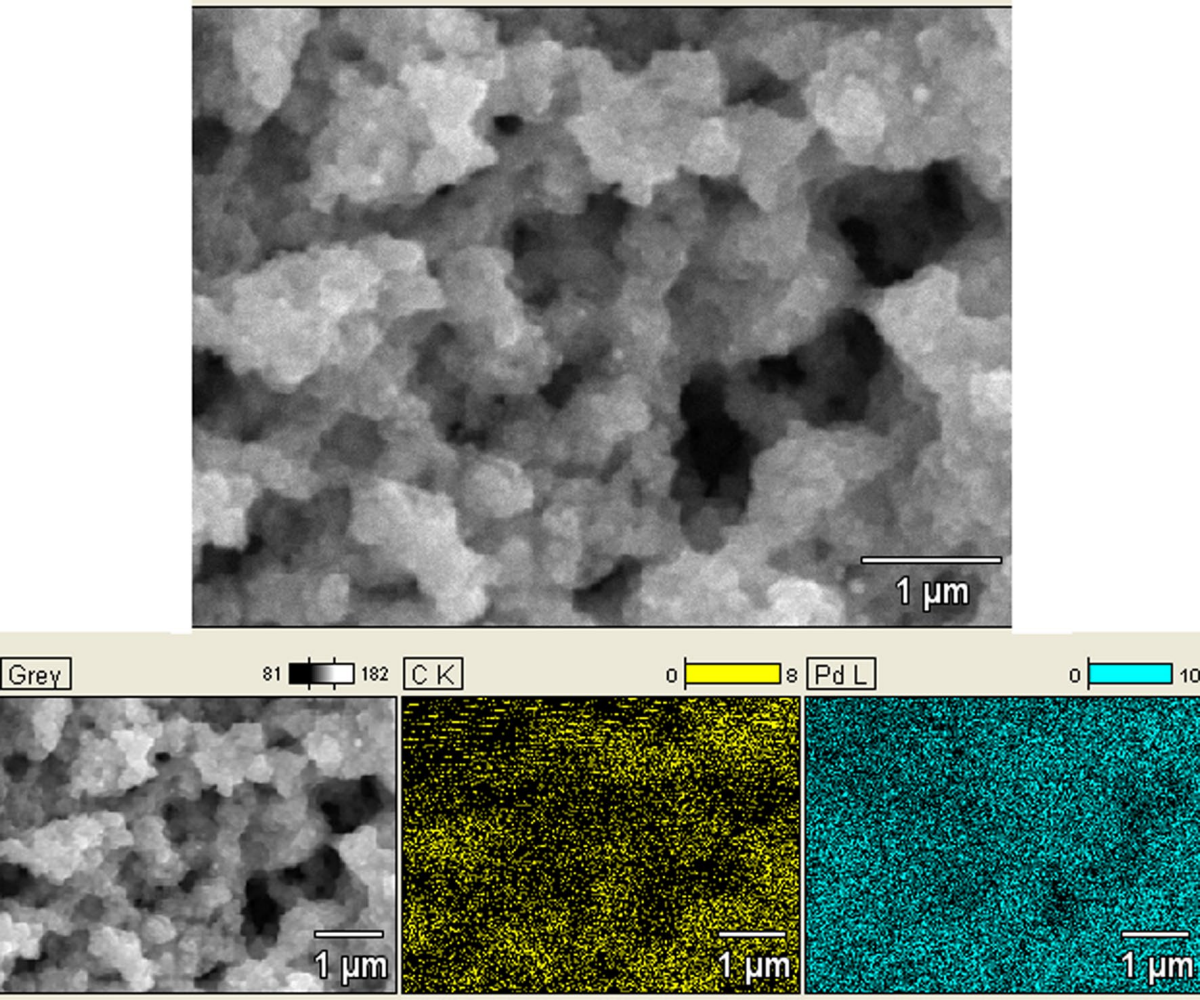
SEM image and EDX analysis of Pd nanoparticles incorporated PAni film

Successful coating of Pd nanoparticle dispersion onto PAni films was confirmed using SEM imaging (Fig. 9). EDX analysis was used to quantify Pd amount in samples. EDX also verified a decent distribution of Pd nanoparticles in PAni films by means of 6.46% in weight and 0.83% by atoms (Fig. 9).

**Fig. 10:**
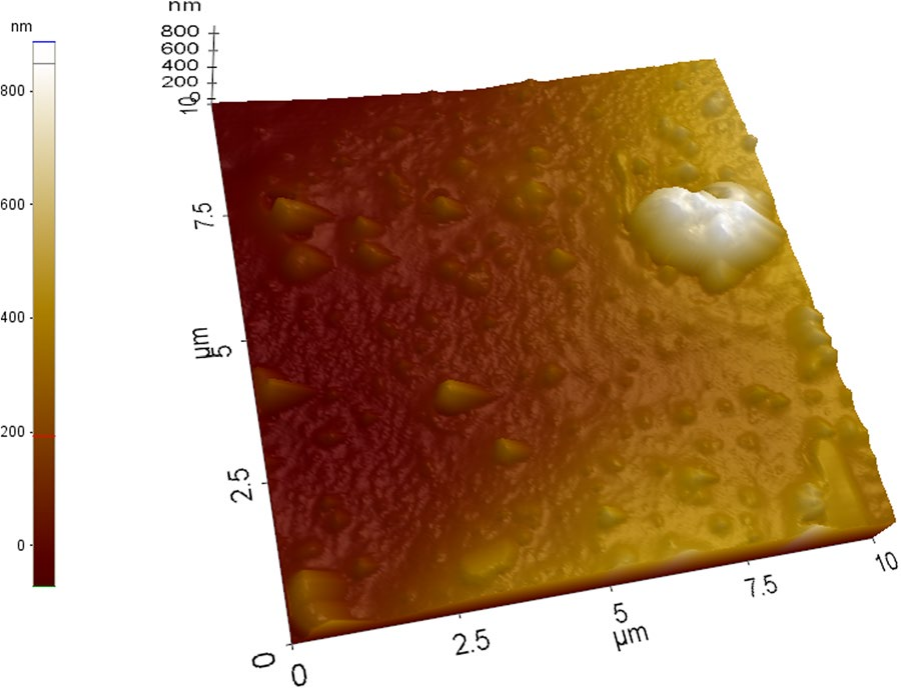
AFM image of Pd nanoparticles incorporated PAni film

When the sensor surface was analyzed by AFM, a homogeneous topographical distribution was observed at most positions with an exception of occasional larger smooth aggregates which could have resulted from polymer–metal complexes. The most prominent topographical feature was the even rough surface consisting of nanostructures (~ 125 nm) arising from PAni film (Fig. 10).

### Impedance analysis for moisture

**Fig. 11:**
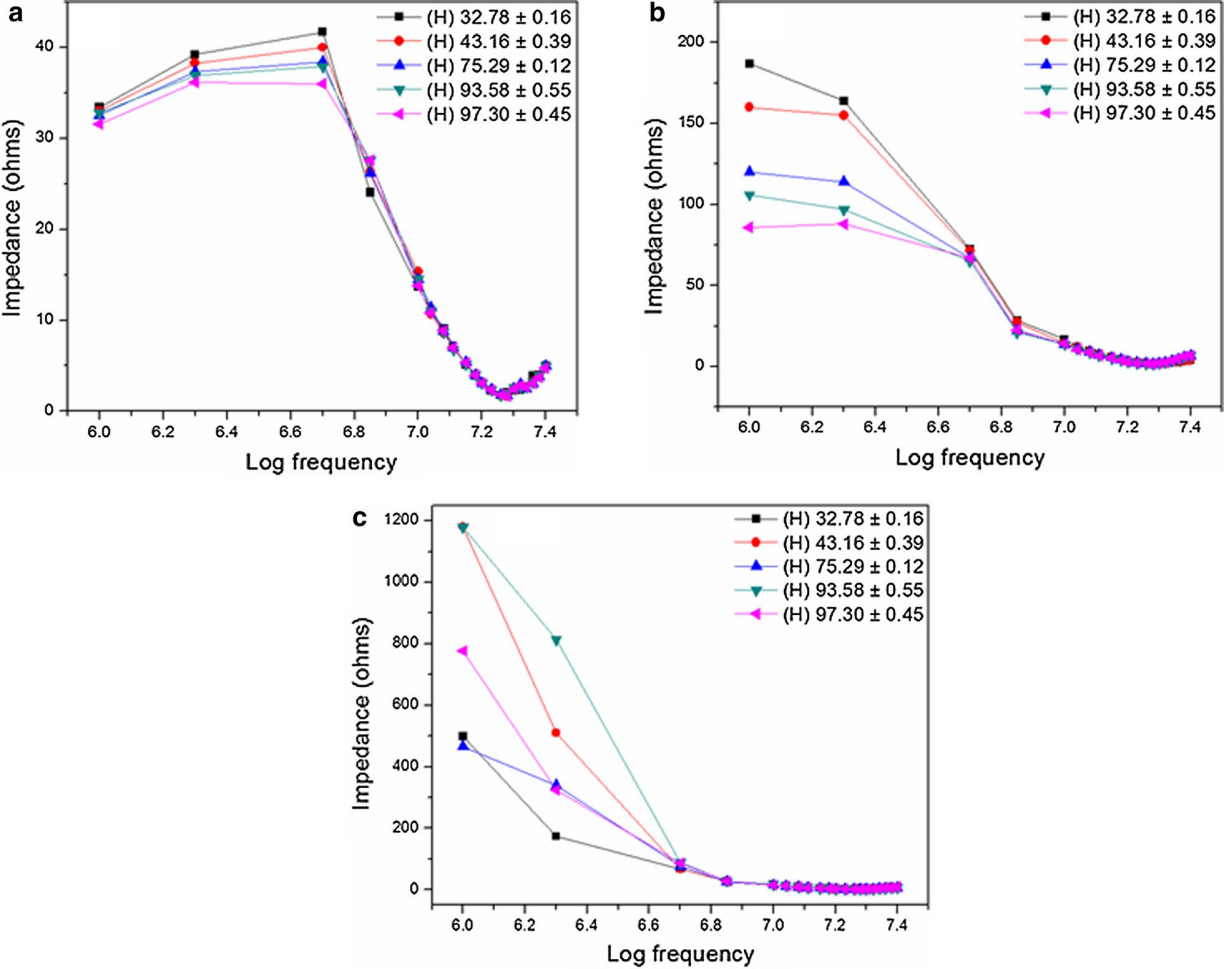
Capacitive behavior of **a** PAni film, **b** Pd incorporated PAni film and **c** Pd only sensors for humidity

According to the impedance data obtained, the PAni sensor exhibits capacitive behavior hence this sensor can be categorized as capacitive type humidity sensor (Fig. 11). PAni film shows the lowest impedance value, while Pd only sensor shows the highest impedance value due to the unavailability of appropriate conductive paths. In the case of PAni, exposure to the water vapor gets PAni protonated (acid base reaction) via an electron hopping assisted by a proton transfer mechanism that results an impedance drop [43, 54, 62, 63]. Meanwhile, Pd incorporated PAni film exhibits impedance in between. Addition of polymer containing Pd nanoparticle solution might be the reason for this observation. Also, the variation of impedance with humidity seems to be almost overlapped in frequencies over 10 MHz range. However, there is a distinct variation, which can be observed in the range of 1–10 MHz.

**Fig. 12:**
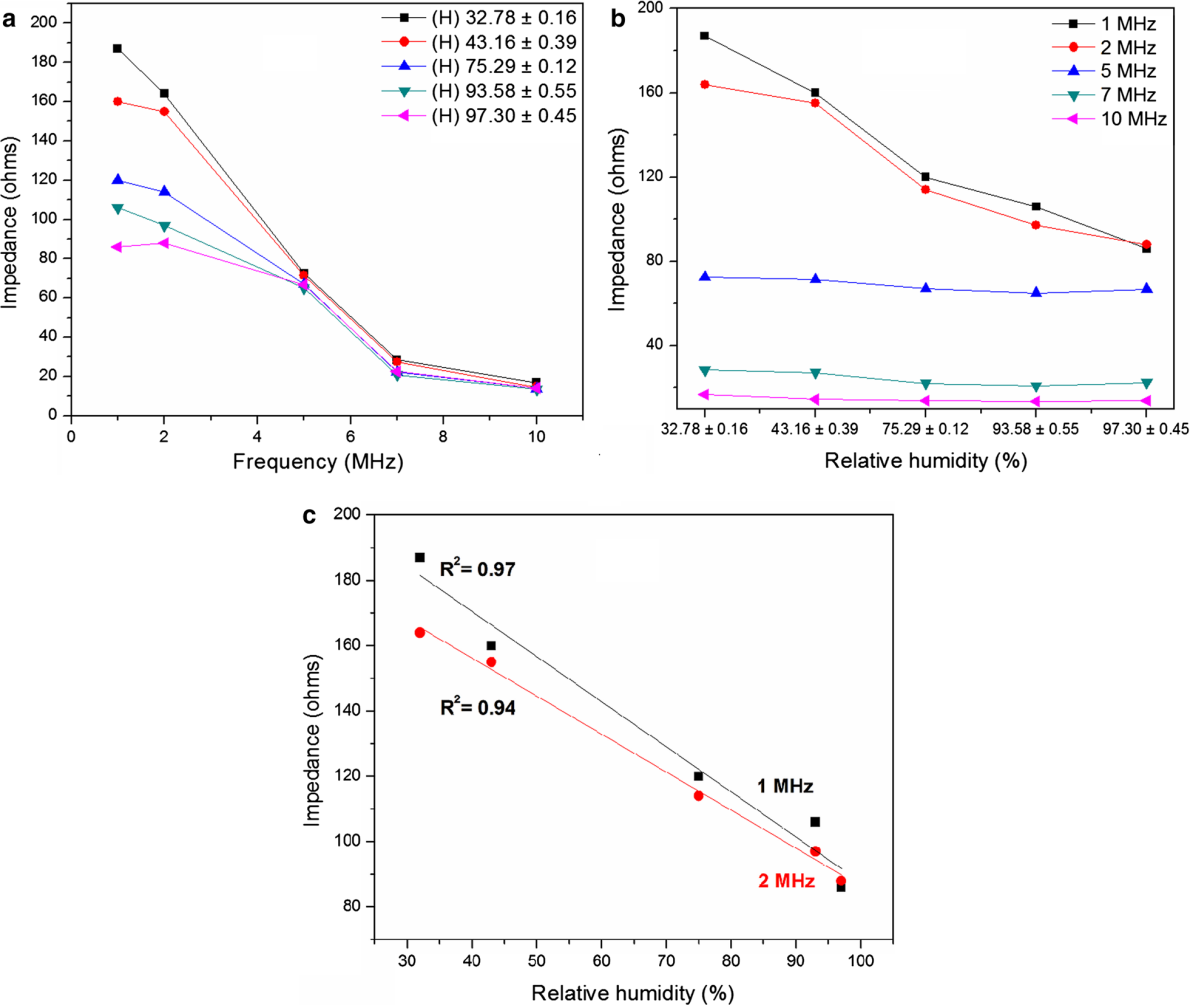
**a** Impedance vs frequency, **b** impedance vs relative humidity in 1–10 MHz frequency domain for Pd incorporated PAni films and **c** linear curve fit at 1 MHz and 2 MHz

In 1–10 MHz region, some variation of impedance with humidity can be seen in PAni film, however the respective variation was marginal in contrast to that of Pd incorporated PAni film. However, a clear dependence of impedance on frequency with a direct correlation can be seen in Pd incorporated PAni film within the 1–10 MHz region (Fig. 12).

According to the Fig. 12a, impedance decreased continuously with frequency under all humidity conditions. Also, the variation of impedance with humidity is distinguishable at the frequencies of 1 and 2 MHz. In both cases, the impedance at highest humidity (97.3%) was less than half of the impedance at lowest humidity (32.8%).

In Fig. 12b, relative humidity is plotted against impedance and it further justified the observation made before. Moreover, the figure indicated that the impedance variation at 1 MHz was much superior and more linear (R^2^ = 0.97) implying that it is more suitable for sensor development in comparison to the sensitivity and linearity at 2 MHz (R^2^ = 0.94) (Fig. 12c).

### Impedance analysis for H_2_

Similar to the humidity sensing experiment, PAni films exhibit a capacitive behavior, hence the possible sensing element can be categorized as a capacitive type sensor.

**Fig. 13:**
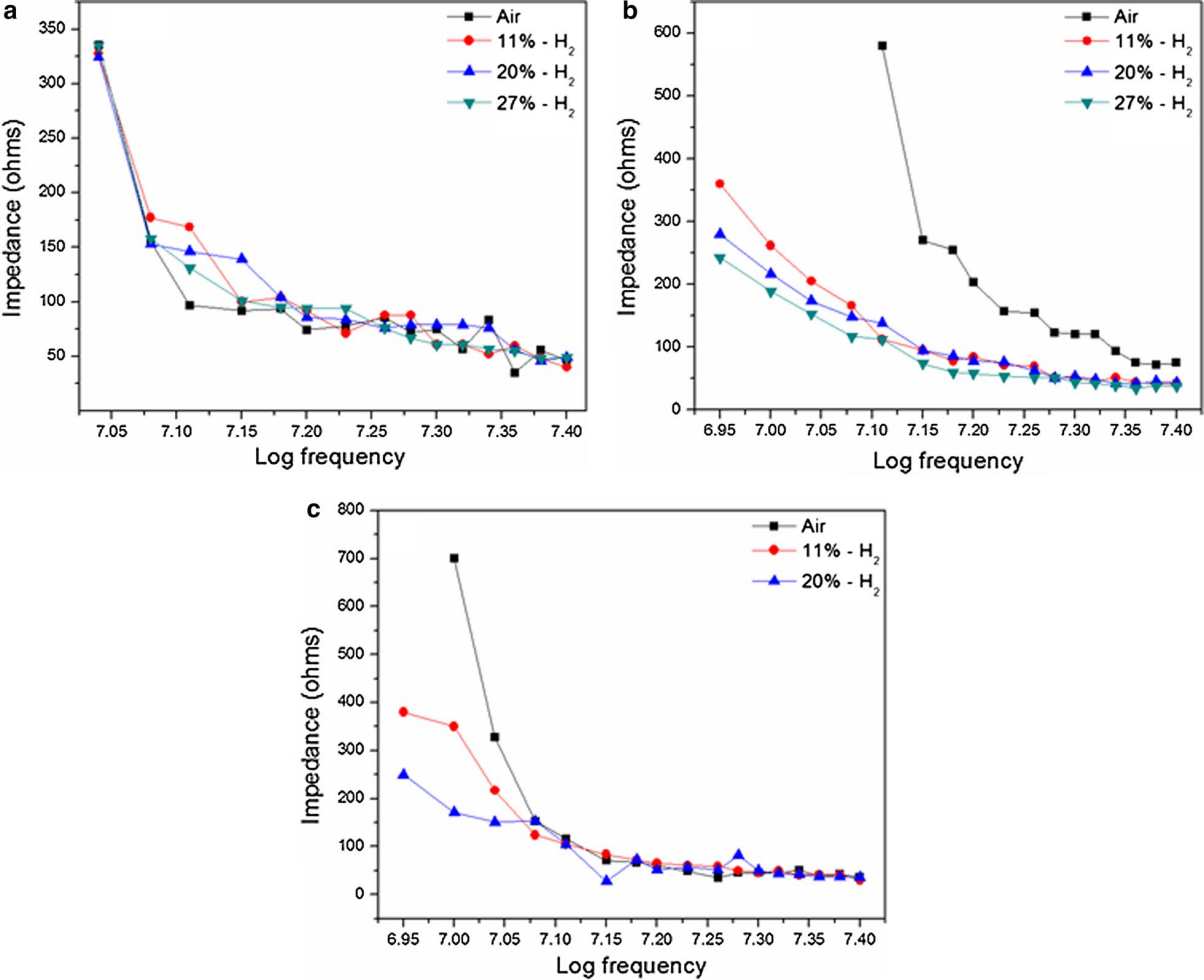
Capacitive behavior of **a** PAni film, **b** Pd incorporated PAni film and **c** Pd only for H_2_ sensing

**Fig. 14:**
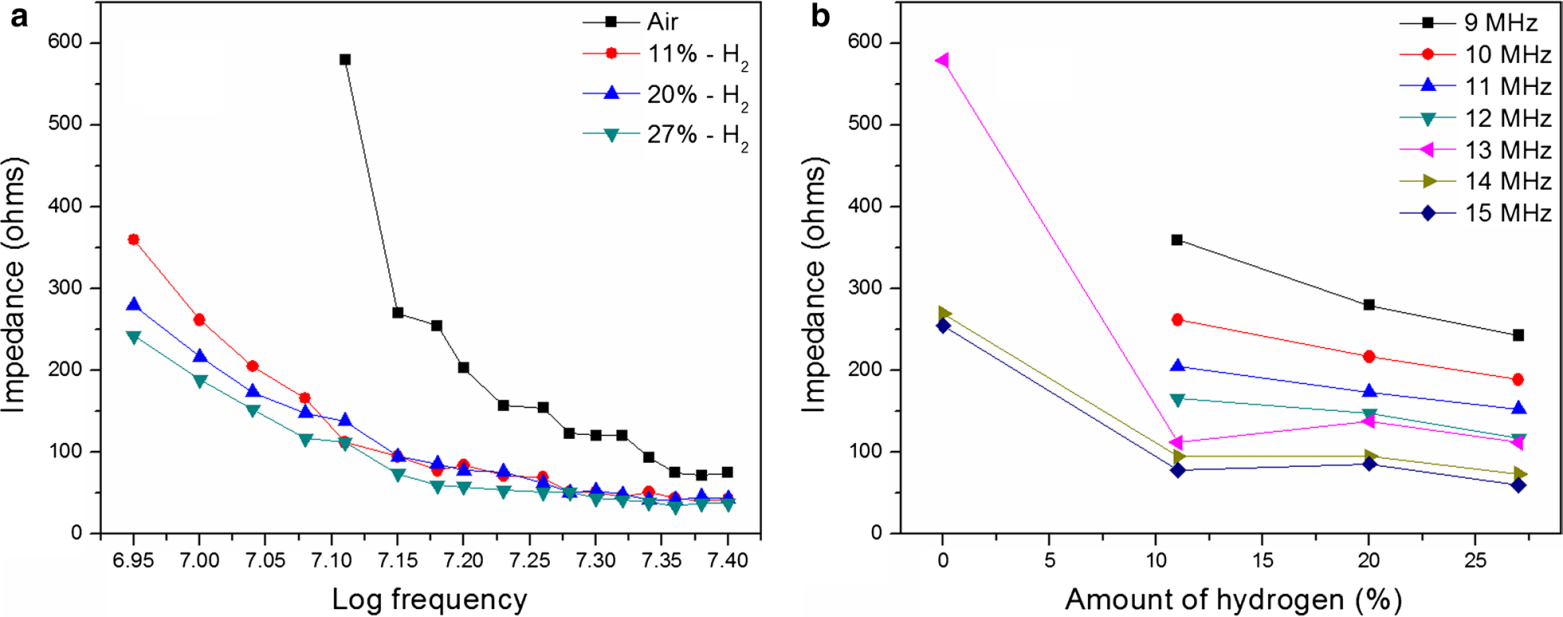
**a** Impedance vs frequency and **b** impedance vs H_2_ percentage in 9–15 MHz frequency domain for Pd incorporated PAni films

Interestingly, the impedance drop in Pd incorporated PAni film and Pd only film was distinguishable. However, it is hard to notice a visible correlation between impedance and frequency for PAni film (Fig. 13). Observations seem somewhat contradictory with some reported literature [64–66]. Presence of humidity in the H_2_ environment may be the reason for such a deviation [54]. Nevertheless, once PAni film was treated with Pd, a significant improvement in sensitivity for H_2_ was observed. Thus, Pd incorporated PAni film exhibited much superior performance towards H_2_ (Fig. 14).

Impedance drop with the elevation of H_2_ concentration and the frequency were clearly observed for Pd incorporated PAni film (Fig. 14). Results shown in Fig. 13b verified the previous observation and it further reveals that frequencies from 9 to 12 MHz were well suited for quantifying the H_2_ levels, due to its steadiness in impedance drop where the regression analysis (R^2^) for linear curve fitting results over 0.90 for all frequencies. However, the sensitivity drop with the increasing frequency must also be taken into consideration in such an instance. Even though the impedance varied in a narrower range in presence of H_2_ at higher frequencies (13–15 MHz), a substantial variation in impedance was found as the sensor was exposed to the H_2_ gas. In detail, at 13 MHz, impedance decreased around 1/5th of its original value in the presence of H_2_ (11%), and it decreased less than half at both frequencies of 14 MHz and 15 MHz (Fig. 14b). Therefore, the Pd incorporated PAni film is well suited for the detection of H_2_ at 13–15 MHz frequency range.

**Fig. 15:**
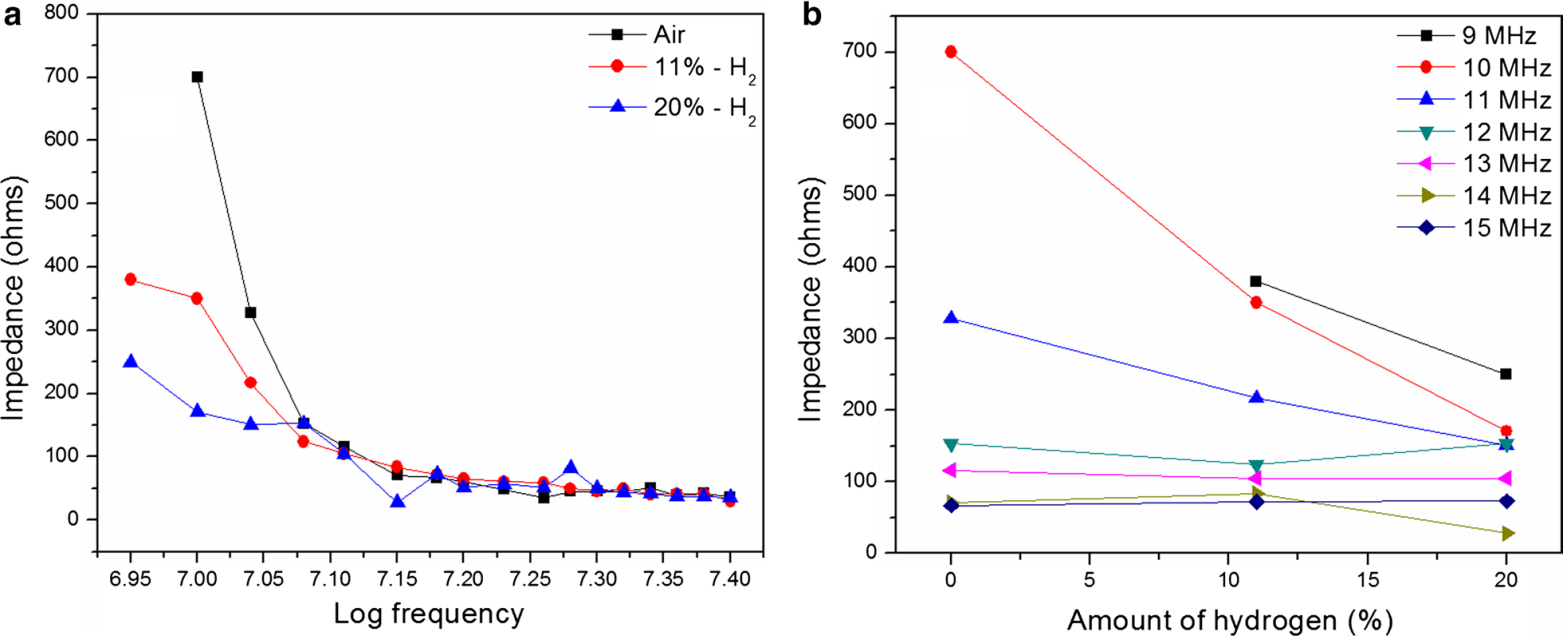
**a** Impedance vs frequency and **b** impedance vs H_2_ percentage in 9–15 MHz frequency domain for Pd only film

Interestingly, Pd only also displays a substantial sensitivity towards H_2_. That is only possible due to the activity of Pd nanoparticles (Fig. 15).

Again, an impedance drop with frequency can be seen for Pd only film. Interestingly, a similar behavior was observed with the increment of H_2_ partial pressure at lower frequencies (9–11 MHz) (Fig. 15). Unlike for Pd incorporated PAni film, H_2_ gas sensitivity is far minimal at other frequencies (Fig. 15b).

The results evidently revealed that Pd nanoparticles were the key component in detecting H_2_ gas. The capability of Pd to adsorb hydrogen and its ability to break H–H bond may be the cause for the impedance drop in the presence of H_2_ [11, 31]. The enhancement of sensor performance inflicted by Pd nanoparticles incorporation into PAni film may be due to the possible spillover of H atoms (found from broken H–H bond) towards the neighboring sites of PAni matrix that facilitates the proton transfer mechanism (earlier described under the “Impedance analysis for H_2_” section) via its conducting pathways.

## Conclusions

This study has shown that PAni is a suitable material for the detection of humidity. Incorporation of Pd to PAni increased the sensitivity for humidity. Importantly, PAni film alone did not exhibit H_2_ sensing properties. Hence, the presence of humidity in H_2_ might be the reason for such observations. Moreover, Pd only also exhibits hydrogen sensing activity and Pd incorporated PAni film shows significant sensing performances towards hydrogen. Fast and easy fabrication and cost-effectiveness would justify the candidacy of Pd incorporated PAni towards sensing both humidity and hydrogen.

## Abbreviations

Pd NPs: palladium nanoparticles
D/F PT: dew/frost point
RH: relative humidity
PAni: polyaniline
ER: epoxy resin
PVP: poly (*N*-vinyl-2-pyrrolidone)
PIPTF: Pd nanoparticles incorporated PAni thin films
PDI: polydispersion index
EDX: energy dispersive x-ray
SEM: scanning electron microscopy
FT-IR: Fourier transform infra-red
